# Two–Photon Absorption Properties and Structure–Property Relationships of Natural 9,10–Anthraquinones: A Curated RI–CC2 Dataset

**DOI:** 10.3390/ijms27010087

**Published:** 2025-12-21

**Authors:** Maciej Spiegel

**Affiliations:** Department of Organic Chemistry and Pharmaceutical Technology, Faculty of Pharmacy, Wroclaw Medical University, Borowska 211A, 50–556 Wroclaw, Poland; maciej.spiegel@umw.edu.pl

**Keywords:** two–photon absorption, anthraquinones, natural products, coupled–cluster theory, photodynamic therapy, RI–CC2, non–linear optics, photosensitisers, computational chemistry, TD–DFT

## Abstract

This work provides the first systematic survey of the two–photon properties of 97 natural 9,10–anthraquinones from plants and fungi. A comprehensive computational dataset of two–photon absorption properties calculated using RI–CC2/aug–cc–pVDZ is presented. Single degenerate photon energies required for two–photon excitation span 491.6–1007.9 nm across the five lowest singlet states, with all S_0_→S_1_ transitions falling within the biological therapeutic window. Remarkably, S_3_ state exhibits systematically enhanced TPA efficiency, with 60% of compounds surpassing 1 GM and achieving a mean cross–section of 29.9 GM–substantially higher than S_1_ (mean: 7.5 GM). Three compounds demonstrate exceptional performance: cynodontin (73.6 GM, S_2_), dermocybin (68.7 GM, S_4_), and morindone (50.7 GM, S_3_). Orbital analysis reveals that these excitations possess high configurational purity and diagnostics validating the single–reference treatment. The observed spatial separation between hole and particle NTOs, combined with extreme transition dipole anisotropy along the molecular long axis, indicates dipolar charge–transfer enhancement. Comprehensive structure–property analysis establishes that strategic modification may maximise TPA cross–sections. Comparison with aqueous–phase calculations for three compounds reveals non–systematic solvent–induced redistributions of TPA activity across excited states, indicating that gas–phase outcomes serve primarily as internal benchmarks and intrinsic descriptors of structure–property relationships rather than quantitative predictors of photoactivity.

## 1. Introduction

Computational quantum chemistry has emerged as an essential tool for predicting molecular properties at the atomic level, delivering insights with impressive efficiency and low cost. One particularly important application lies in non–linear optics, specifically two–photon absorption (TPA), where molecules simultaneously absorb two lower–energy photons rather than one higher–energy photon. This process offers superior spatial resolution and deeper tissue penetration, making it valuable for technologies such as photocatalysis [1,2], photovoltaics [3,4], bioimaging [5,6,7], and photodynamic therapy [8,9,10,11]. Building on this potential, researchers have increasingly focused on understanding and predicting TPA properties from first principles.

Amongst the diverse chemical families explored for TPA applications, anthraquinones have attracted attention due to their tuneable non–linear optical properties and structural versatility. Recent work has demonstrated that anthraquinone–centred photosensitisers with aggregation–induced emission characteristics can enable efficient type I photodynamic therapy, exhibiting substantial TPA cross–sections ( $\sigma_{TPA}$ ) under near–infrared (NIR) illumination [11]. Individual naturally occurring molecules such as soranjidiol [12], aloe–emodin [13], and rubiadin [14] also show encouraging one– and two–photon absorption profiles as heavy–atom–free photosensitisers, albeit with somewhat lower efficiencies than heavy–atom–containing dyes. The core anthraquinone structure–aromatic rings symmetrically fused around a benzoquinone motif–provides a versatile scaffold for fine–tuning optical responses, particularly for NIR–II PDT in hypoxic tumour environments [15,16].

**Figure 1:**
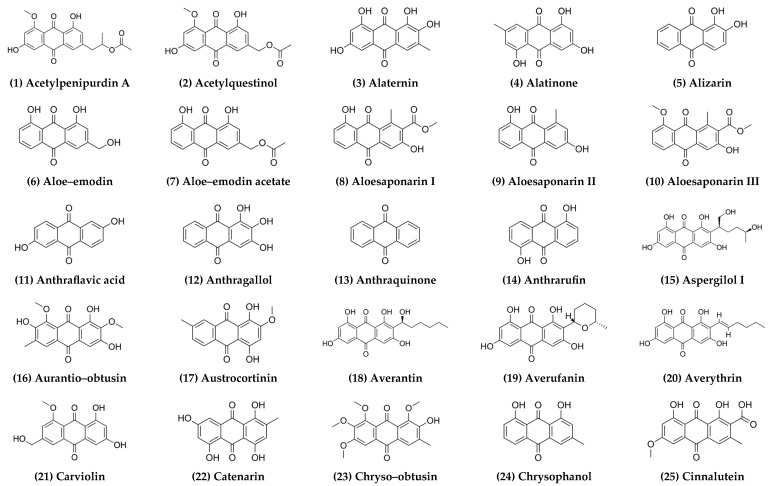

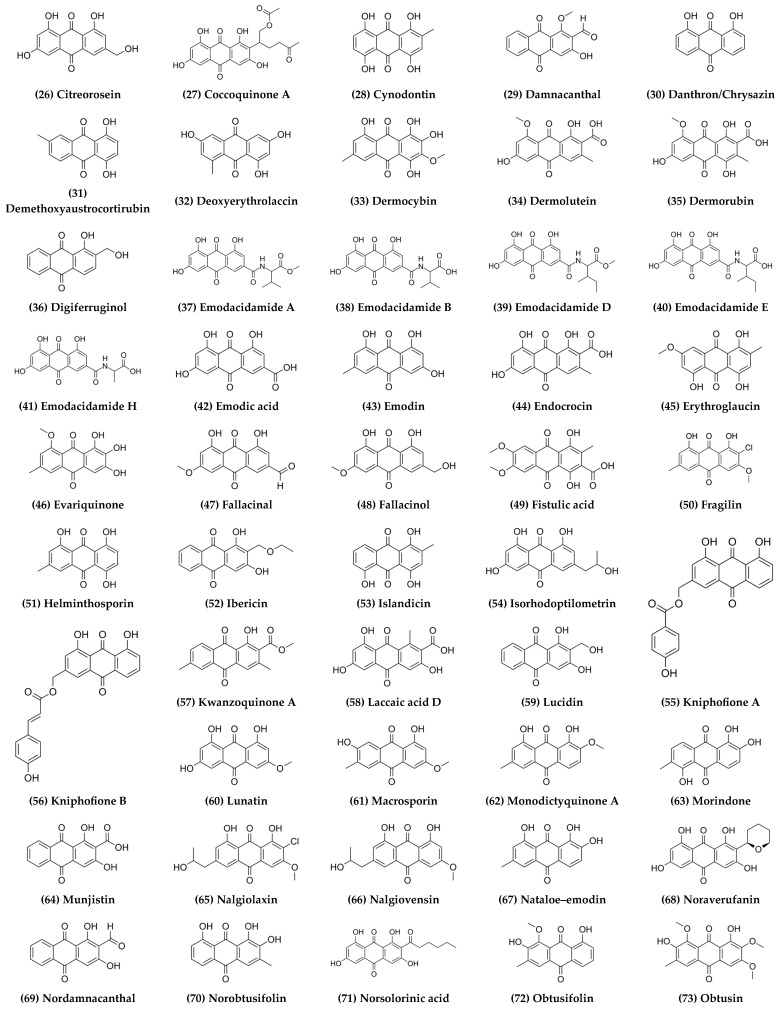

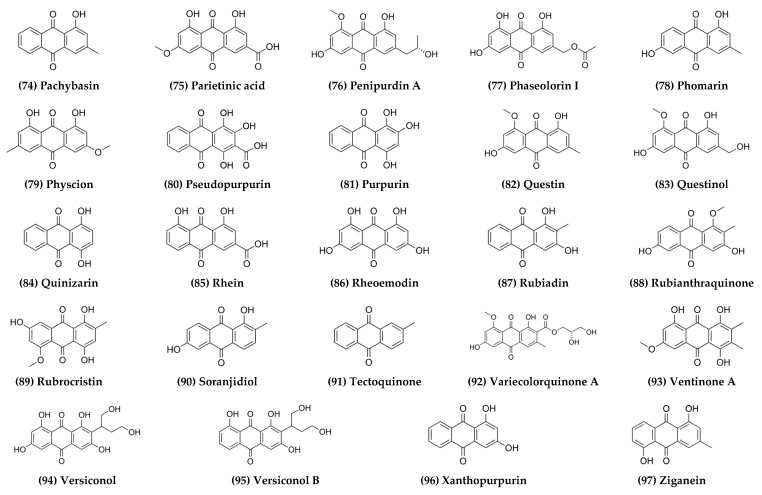
Graphical representation of the 9,10–anthraquinones considered in this study.

Despite this promise, predicting TPA properties in naturally occurring anthraquinones remains challenging, largely due to the scarcity of experimental data on their non–linear optical behaviour. This shortage of experimental benchmarks necessitates reliance on computationally demanding methods, limiting rapid exploration despite encouraging validations such as accurate absorption wavelength predictions. Systematic studies remain absent from the literature, and so to address this gap, the present study provides the first comprehensive computational dataset of TPA properties for 97 natural anthraquinones from plants and fungi (Figure 1), calculated using the RI–CC2 method–a level of theory widely regarded as a benchmark for TPA predictions offering a favourable balance between accuracy and computational cost. This curated dataset aims to facilitate identification of high–performance natural scaffolds and guide future experimental validation efforts in two–photon–activated therapeutic applications.

## 2. Results and Discussion

The computed raw two–photon properties for the studied anthraquinone derivatives are compiled in Table 1

### 2.1. Validation of Computational Approach

To validate the suitability of the single–reference CC2 method for this large series of anthraquinone derivatives, the D_1_ diagnostic, a commonly used measure of multi–reference character in the ground–state wave function [17], was evaluated.

The calculated D_1_ values range from a minimum of 0.0624 (ziganein) to a maximum of 0.1268 (cynodontin), with a mean value of 0.1069 across all 97 molecules. Whilst the strict single–reference regime traditionally flags D_1_ values ≥ 0.05 as concerning [17,18], this limit is often considered overly restrictive. Köhn et al. [19] proposed a limit of 0.10–0.15 as a more appropriate threshold for the RI–CC2. Given that all D_1_ diagnostics in this study fall within or below the acceptable threshold range, the calculated properties related to two–photon absorption can be considered quantitatively reliable, allowing direct comparison of both absolute values and relative trends. Moreover, the consistency of the results is further supported by the low standard deviation (±0.0111), reinforcing the assumption that systematic errors are uniform across the dataset.

**Table 1:** Calculated two–photon absorption properties for the studied 9,10–anthraquinones.

Molecule	D_1_	S*_i_*	$\lambda_{1\omega }$	δ_TPA_	σ_TPA_
Acetylpenipurdin A	0.1039	*1*	819.2	4.52	0.02
		*2*	808.2	702.57	3.58
		*3*	745.3	0.24	0.00
		*4*	713.4	4434.21	28.96
		*5*	623.2	1341.54	11.48
Acetylquestinol	0.1034	*1*	814.7	0.35	0.00
		*2*	797.0	500.10	2.62
		*3*	743.8	0.08	0.00
		*4*	709.6	4422.10	29.19
		*5*	615.9	1362.08	11.94
Alaternin	0.1105	*1*	811.2	6.77	0.03
		*2*	799.4	2704.07	14.07
		*3*	770.6	2333.57	13.06
		*4*	661.5	1776.88	13.50
		*5*	644.9	0.81	0.01
Alatinone	0.1078	*1*	775.8	183.34	1.01
		*2*	767.3	276.68	1.56
		*3*	766.2	2875.17	16.28
		*4*	693.6	0.01	0.00
		*5*	639.7	2343.84	19.04
Alizarin	0.0983	*1*	805.9	7.05	0.04
		*2*	799.8	2703.61	14.05
		*3*	690.5	0.17	0.00
		*4*	642.5	906.46	7.30
		*5*	589.0	602.54	5.77
Aloe–emodin	0.1080	*1*	817.9	0.37	0.00
		*2*	799.4	87.38	0.45
		*3*	725.7	5192.03	32.77
		*4*	672.9	0.40	0.00
		*5*	614.8	962.03	8.46
Aloe–emodin acetate	0.1078	*1*	821.3	0.45	0.00
		*2*	802.4	93.03	0.48
		*3*	731.0	5242.35	32.61
		*4*	675.5	0.66	0.00
		*5*	620.3	1028.32	8.88
Aloesaponarin I	0.1020	*1*	802.6	32.93	0.17
		*2*	771.7	675.49	3.77
		*3*	717.9	120.59	0.78
		*4*	688.7	2937.44	20.59
		*5*	611.5	2138.08	19.00
Aloesaponarin II	0.1001	*1*	797.9	0.94	0.00
		*2*	776.5	967.92	5.34
		*3*	720.8	0.26	0.00
		*4*	669.5	3155.46	23.40
		*5*	596.9	586.63	5.47
Aloesaponarin III	0.0922	*1*	817.3	10.54	0.05
		*2*	746.6	45.35	0.27
		*3*	714.5	85.11	0.55
		*4*	678.4	1411.71	10.20
		*5*	619.0	415.52	3.61
Anthraflavic acid	0.0858	*1*	781.2	0.20	0.00
		*2*	733.7	0.00	0.00
		*3*	687.9	2131.54	14.98
		*4*	611.9	0.00	0.00
		*5*	573.7	269.59	2.72
Anthragallol	0.1001	*1*	793.7	0.17	0.00
		*2*	764.9	2046.71	11.63
		*3*	722.6	1139.20	7.25
		*4*	684.2	0.19	0.00
		*5*	587.9	745.63	7.17
Anthraquinone	0.0795	*1*	794.6	0.17	0.00
		*2*	587.9	542.80	5.22
		*3*	579.5	0.00	0.00
		*4*	573.8	604.32	6.10
		*5*	491.6	0.00	0.00
Anthrarufin	0.1040	*1*	790.0	0.00	0.00
		*2*	752.1	4040.26	23.74
		*3*	701.6	0.00	0.00
		*4*	609.2	2032.16	18.20
		*5*	518.2	1408.38	17.43
Aspergilol I	0.1140	*1*	840.8	255.27	1.20
		*2*	800.1	0.26	0.00
		*3*	718.0	3736.56	24.10
		*4*	686.7	5282.10	37.24
		*5*	646.5	5.93	0.05
Aurantio–obtusin	0.1018	*1*	816.2	0.84	0.00
		*2*	759.2	2194.55	12.66
		*3*	735.0	35.77	0.22
		*4*	720.4	1946.11	12.46
		*5*	660.8	1355.10	10.32
Austrocortinin	0.1230	*1*	921.6	2844.81	11.13
		*2*	760.5	0.64	0.00
		*3*	692.7	0.32	0.00
		*4*	668.3	1808.94	13.47
		*5*	618.1	879.17	7.65
Averantin	0.1134	*1*	831.7	70.41	0.34
		*2*	802.1	0.67	0.00
		*3*	710.7	4764.41	31.35
		*4*	680.9	3100.49	22.23
		*5*	651.4	9.12	0.07
Averufanin	0.1129	*1*	831.4	136.03	0.65
		*2*	799.9	0.30	0.00
		*3*	711.6	4588.38	30.12
		*4*	683.6	4059.58	28.88
		*5*	648.7	7.68	0.06
Averythrin	0.1131	*1*	843.7	6447.95	30.11
		*2*	821.3	9659.53	47.61
		*3*	801.6	3.22	0.02
		*4*	695.7	2681.00	18.41
		*5*	659.8	0.86	0.01
Carviolin	0.1033	*1*	807.4	1.01	0.01
		*2*	789.8	431.52	2.30
		*3*	742.3	0.16	0.00
		*4*	698.3	4244.03	28.93
		*5*	627.7	1462.47	12.34
Catenarin	0.1211	*1*	931.6	1329.60	5.09
		*2*	761.1	1.83	0.01
		*3*	747.9	5814.81	34.55
		*4*	664.7	0.60	0.00
		*5*	649.4	2822.61	22.25
Chryso–obtusin	0.0911	*1*	801.4	60.20	0.31
		*2*	742.2	1069.68	6.46
		*3*	717.2	1563.23	10.10
		*4*	676.4	1393.30	10.12
		*5*	643.5	1304.68	10.47
Chrysophanol/	0.1077	*1*	818.9	0.27	0.00
Chrysophanic acid		*2*	799.1	91.72	0.48
		*3*	726.0	4984.13	31.43
		*4*	672.5	0.41	0.00
		*5*	619.2	986.47	8.55
Cinnalutein	0.1137	*1*	822.7	127.31	0.63
		*2*	807.1	1.55	0.01
		*3*	722.0	7628.14	48.65
		*4*	665.1	28.50	0.21
		*5*	652.1	2157.09	16.86
Citreorosein/	0.1100	*1*	808.1	4.30	0.02
ω–hydroxyemodin		*2*	804.0	1.43	0.01
		*3*	711.7	5318.54	34.90
		*4*	663.2	1.72	0.01
		*5*	640.0	1604.72	13.03
Coccoquinone A	0.1137	*1*	837.8	9.82	0.05
		*2*	798.0	0.30	0.00
		*3*	730.1	5721.08	35.68
		*4*	685.1	2557.60	18.11
		*5*	665.4	1.54	0.01
Cynodontin	0.1268	*1*	1007.9	0.94	0.00
		*2*	815.5	14,730.65	73.63
		*3*	746.5	3.11	0.02
		*4*	666.0	2326.75	17.44
		*5*	659.3	0.18	0.00
Damnacanthal	0.0949	*1*	814.9	29.83	0.15
		*2*	751.9	30.54	0.18
		*3*	738.6	196.86	1.20
		*4*	667.8	62.68	0.47
		*5*	614.5	1395.54	12.29
Danthron/	0.1072	*1*	824.5	0.27	0.00
Chrysazin		*2*	797.0	123.09	0.64
		*3*	730.1	4651.64	29.01
		*4*	674.9	0.41	0.00
		*5*	608.1	702.32	6.31
Demethoxyaustrocortirubin	0.1161	*1*	921.5	2323.61	9.10
		*2*	777.3	0.72	0.00
		*3*	703.2	0.19	0.00
		*4*	635.2	1157.94	9.54
		*5*	601.7	1264.35	11.61
Deoxyerythrolaccin	0.1022	*1*	786.8	115.16	0.62
		*2*	785.1	518.09	2.79
		*3*	713.3	0.21	0.00
		*4*	667.7	2812.95	20.98
		*5*	612.9	1288.18	11.40
Dermocybin	0.1267	*1*	911.0	1784.23	7.15
		*2*	795.9	1539.92	8.08
		*3*	756.4	74.58	0.43
		*4*	717.9	10,653.97	68.71
		*5*	647.1	1.78	0.01
Dermolutein	0.1050	*1*	819.6	5.98	0.03
		*2*	805.9	570.77	2.92
		*3*	745.6	3.22	0.02
		*4*	716.6	5460.42	35.35
		*5*	623.8	1626.54	13.89
Dermorubin	0.1200	*1*	946.2	2603.10	9.66
		*2*	819.4	2.16	0.01
		*3*	732.1	5391.86	33.44
		*4*	706.1	1.12	0.01
		*5*	632.2	2758.02	22.94
Digiferruginol	0.0962	*1*	806.6	0.26	0.00
		*2*	766.0	1966.67	11.14
		*3*	706.2	0.16	0.00
		*4*	609.1	1021.45	9.15
		*5*	589.0	694.04	6.65
Emodacidamide A	0.1107	*1*	821.7	11.89	0.06
		*2*	806.7	3.81	0.02
		*3*	719.9	6497.67	41.67
		*4*	667.8	51.52	0.38
		*5*	641.2	1785.75	14.44
Emodacidamide B	0.1106	*1*	821.8	0.06	0.00
		*2*	807.9	6.48	0.03
		*3*	720.5	6355.87	40.70
		*4*	667.7	24.42	0.18
		*5*	641.7	1758.23	14.19
Emodacidamide D	0.0648	*1*	813.2	5.17	0.03
		*2*	810.5	2.37	0.01
		*3*	726.4	5794.07	36.50
		*4*	672.2	12.82	0.09
		*5*	653.6	1393.02	10.84
Emodacidamide E	0.1109	*1*	813.1	7.01	0.04
		*2*	810.5	3.82	0.02
		*3*	726.0	5715.57	36.04
		*4*	673.1	9.92	0.07
		*5*	652.8	1443.39	11.26
Emodacidamide H	0.1106	*1*	821.9	6.13	0.03
		*2*	807.1	11.60	0.06
		*3*	720.1	6134.89	39.33
		*4*	667.4	27.12	0.20
		*5*	641.7	1743.17	14.07
Emodic acid	0.1100	*1*	818.3	0.48	0.00
		*2*	807.8	56.90	0.29
		*3*	724.3	5262.50	33.35
		*4*	671.4	0.60	0.00
		*5*	643.3	1665.52	13.38
Emodin	0.0636	*1*	807.4	11.54	0.06
		*2*	804.9	1.45	0.01
		*3*	712.2	5139.67	33.69
		*4*	661.7	0.57	0.00
		*5*	639.7	1549.92	12.59
Endocrocin	0.1110	*1*	816.9	15.59	0.08
		*2*	809.7	4.41	0.02
		*3*	722.9	6375.97	40.56
		*4*	667.5	29.26	0.22
		*5*	649.6	1739.11	13.70
Erythroglaucin	0.1223	*1*	927.6	1054.18	4.07
		*2*	757.0	1162.81	6.75
		*3*	756.3	6502.67	37.79
		*4*	661.9	0.51	0.00
		*5*	650.1	2761.10	21.72
Evariquinone	0.1040	*1*	797.5	2.40	0.01
		*2*	768.7	1858.28	10.45
		*3*	750.3	1701.99	10.05
		*4*	728.7	4.80	0.03
		*5*	681.9	3340.26	23.88
Fallacinal	0.1127	*1*	815.6	280.62	1.40
		*2*	813.0	343.09	1.73
		*3*	720.3	5873.96	37.63
		*4*	692.4	0.16	0.00
		*5*	667.1	1.10	0.01
Fallacinol	0.1126	*1*	811.5	101.17	0.51
		*2*	799.8	0.23	0.00
		*3*	709.2	6360.77	42.04
		*4*	660.0	0.50	0.00
		*5*	641.6	2108.02	17.02
Fistulic acid	0.1186	*1*	918.2	2588.63	10.21
		*2*	795.7	2650.59	13.92
		*3*	769.4	4.22	0.02
		*4*	691.7	0.24	0.00
		*5*	627.0	3312.61	28.01
Fragilin	0.1125	*1*	815.0	21.12	0.11
		*2*	805.3	0.33	0.00
		*3*	748.6	3948.85	23.42
		*4*	712.7	5186.56	33.94
		*5*	666.5	0.39	0.00
Helminthosporin	0.1205	*1*	930.4	1297.39	4.98
		*2*	772.7	2.11	0.01
		*3*	747.7	6813.54	40.52
		*4*	672.3	0.43	0.00
		*5*	636.4	1817.40	14.91
Ibericin	0.1022	*1*	794.9	1.20	0.01
		*2*	770.6	1561.63	8.74
		*3*	696.7	39.09	0.27
		*4*	680.6	1395.74	10.02
		*5*	586.2	1014.65	9.82
Islandicin	0.1197	*1*	934.3	1299.05	4.95
		*2*	773.1	0.98	0.01
		*3*	742.7	7069.41	42.61
		*4*	672.7	0.41	0.00
		*5*	638.6	2495.68	20.34
Isorhodoptilometrin	0.1104	*1*	811.6	43.69	0.22
		*2*	806.0	2.11	0.01
		*3*	717.9	5766.25	37.20
		*4*	664.5	1.01	0.01
		*5*	643.7	1610.35	12.92
Kniphofione A	0.1080	*1*	823.7	0.83	0.00
		*2*	803.3	88.50	0.46
		*3*	731.1	5306.66	33.01
		*4*	674.7	0.53	0.00
		*5*	621.5	1253.75	10.79
Kniphofione B	0.1079	*1*	824.3	0.40	0.00
		*2*	803.9	113.00	0.58
		*3*	731.3	5291.89	32.89
		*4*	674.5	0.60	0.00
		*5*	623.9	1530.50	13.07
Kwanzoquinone A	0.0991	*1*	809.4	22.43	0.11
		*2*	777.4	2079.37	11.44
		*3*	709.8	3.36	0.02
		*4*	636.8	2345.92	19.23
		*5*	600.9	751.17	6.92
Laccaic acid D	0.1046	*1*	798.1	131.90	0.69
		*2*	784.3	208.25	1.13
		*3*	710.1	81.67	0.54
		*4*	686.1	2346.99	16.57
		*5*	629.0	1732.78	14.56
Lucidin	0.1019	*1*	795.2	1.33	0.01
		*2*	767.6	1417.04	7.99
		*3*	695.7	17.41	0.12
		*4*	665.9	1352.11	10.14
		*5*	587.3	929.12	8.95
Lunatin	0.1135	*1*	824.2	119.31	0.58
		*2*	794.0	0.44	0.00
		*3*	697.9	5528.08	37.73
		*4*	652.0	12.64	0.10
		*5*	646.7	2212.90	17.59
Macrosporin	0.1037	*1*	789.6	0.20	0.00
		*2*	776.5	2163.40	11.93
		*3*	689.7	0.17	0.00
		*4*	669.8	1341.42	9.94
		*5*	604.6	188.17	1.71
Monodictyquinone A	0.1125	*1*	826.1	3276.37	15.96
		*2*	813.2	13.69	0.07
		*3*	750.4	5430.69	32.06
		*4*	664.1	0.58	0.00
		*5*	645.9	1035.96	8.25
Morindone	0.1077	*1*	826.5	435.02	2.12
		*2*	766.3	0.96	0.01
		*3*	748.7	8542.95	50.66
		*4*	687.6	0.48	0.00
		*5*	668.0	1844.61	13.74
Munjistin	0.1059	*1*	802.4	3.84	0.02
		*2*	792.8	956.58	5.06
		*3*	696.7	0.17	0.00
		*4*	624.6	1605.66	13.68
		*5*	592.8	1499.93	14.19
Nalgiolaxin	0.1130	*1*	819.3	67.07	0.33
		*2*	805.8	0.32	0.00
		*3*	749.5	4403.30	26.06
		*4*	717.9	5486.41	35.38
		*5*	668.3	0.81	0.01
Nalgiovensin	0.1127	*1*	814.8	34.26	0.17
		*2*	801.7	0.40	0.00
		*3*	716.5	6983.25	45.21
		*4*	661.7	1.51	0.01
		*5*	644.3	1889.70	15.13
Nataloe–emodin	0.1082	*1*	821.2	0.50	0.00
		*2*	802.2	1633.84	8.44
		*3*	760.2	3885.23	22.35
		*4*	654.8	0.65	0.01
		*5*	650.4	1065.08	8.37
Noraverufanin	0.1129	*1*	830.8	117.70	0.57
		*2*	800.3	0.66	0.00
		*3*	710.1	4737.38	31.23
		*4*	682.4	3755.63	26.81
		*5*	649.0	7.43	0.06
Nordamnacanthal	0.1050	*1*	805.6	1.09	0.01
		*2*	786.1	649.14	3.49
		*3*	698.7	0.28	0.00
		*4*	656.1	0.29	0.00
		*5*	635.5	1675.02	13.79
Norobtusifolin	0.1081	*1*	827.8	8.97	0.04
		*2*	813.5	2009.56	10.09
		*3*	765.2	2783.54	15.80
		*4*	667.7	1781.25	13.28
		*5*	657.3	3.44	0.03
Norsolorinic acid	0.1147	*1*	854.6	16.67	0.08
		*2*	807.9	0.26	0.00
		*3*	707.8	5001.75	33.19
		*4*	654.6	2655.17	20.60
		*5*	595.0	163.75	1.54
Obtusifolin	0.0977	*1*	832.3	1.38	0.01
		*2*	766.5	1802.77	10.20
		*3*	745.7	70.95	0.42
		*4*	697.0	1602.33	10.96
		*5*	609.6	993.56	8.89
Obtusin	0.1042	*1*	829.7	152.69	0.74
		*2*	782.0	2510.97	13.65
		*3*	748.9	2187.05	12.96
		*4*	740.9	294.07	1.78
		*5*	686.0	2444.29	17.26
Pachybasin	0.0974	*1*	800.9	0.33	0.00
		*2*	766.1	1491.13	8.45
		*3*	707.8	0.15	0.00
		*4*	607.6	900.42	8.11
		*5*	586.9	671.96	6.49
Parietinic acid	0.1127	*1*	814.5	173.62	0.87
		*2*	813.0	252.68	1.27
		*3*	723.3	6309.83	40.09
		*4*	669.6	0.76	0.01
		*5*	649.0	2406.59	18.99
Penipurdin A	0.1037	*1*	814.5	7.76	0.04
		*2*	801.4	703.55	3.64
		*3*	744.4	0.05	0.00
		*4*	709.2	4519.22	29.87
		*5*	619.0	1406.06	12.20
Phaseolorin I	0.1100	*1*	810.1	7.20	0.04
		*2*	807.3	0.74	0.00
		*3*	717.7	5340.03	34.47
		*4*	665.6	1.87	0.01
		*5*	643.5	1529.50	12.28
Phomarin	0.1003	*1*	789.2	1.03	0.01
		*2*	774.8	902.52	5.00
		*3*	706.2	0.19	0.00
		*4*	664.2	3063.92	23.08
		*5*	589.6	450.72	4.31
Physcion/	0.1124	*1*	812.2	61.88	0.31
Parietin		*2*	800.9	0.39	0.00
		*3*	711.3	6245.00	41.04
		*4*	659.0	0.60	0.00
		*5*	641.2	1947.40	15.74
Pseudopurpurin	0.1219	*1*	905.6	1932.61	7.83
		*2*	760.1	0.50	0.00
		*3*	676.2	3037.97	22.09
		*4*	674.6	115.67	0.84
		*5*	627.0	1637.58	13.85
Purpurin	0.1182	*1*	909.5	2113.25	8.49
		*2*	760.8	0.49	0.00
		*3*	687.0	0.28	0.00
		*4*	672.9	1344.72	9.87
		*5*	608.1	1035.95	9.31
Questin	0.1035	*1*	810.5	0.49	0.00
		*2*	796.5	535.85	2.81
		*3*	742.3	0.14	0.00
		*4*	704.9	4227.77	28.29
		*5*	614.6	1353.80	11.91
Questinol	0.1034	*1*	811.3	0.49	0.00
		*2*	793.8	468.87	2.47
		*3*	742.2	0.11	0.00
		*4*	705.6	4393.37	29.34
		*5*	613.1	1383.28	12.23
Quinizarin	0.1156	*1*	918.9	2115.42	8.33
		*2*	775.3	0.65	0.00
		*3*	702.1	0.16	0.00
		*4*	627.4	1144.31	9.66
		*5*	590.4	518.43	4.94
Rhein	0.1075	*1*	834.6	0.38	0.00
		*2*	806.2	197.83	1.01
		*3*	740.9	4888.24	29.60
		*4*	681.7	0.46	0.00
		*5*	605.8	684.60	6.20
Rheoemodin	0.1112	*1*	818.9	12.03	0.06
		*2*	797.6	0.27	0.00
		*3*	699.8	4666.08	31.67
		*4*	654.6	4.99	0.04
		*5*	647.6	1880.33	14.90
Rubiadin	0.1002	*1*	790.0	0.49	0.00
		*2*	767.4	1471.25	8.30
		*3*	703.5	7.73	0.05
		*4*	690.6	1588.35	11.07
		*5*	587.9	821.32	7.90
Rubianthraquinone	0.0852	*1*	855.7	5.71	0.03
		*2*	738.2	6.93	0.04
		*3*	698.3	867.13	5.91
		*4*	639.3	1096.39	8.92
		*5*	599.0	385.91	3.58
Rubrocristin	0.1195	*1*	948.8	2327.63	8.60
		*2*	821.8	0.62	0.00
		*3*	724.1	4331.60	27.46
		*4*	706.4	0.26	0.00
		*5*	627.4	2947.10	24.89
Soranjidiol	0.0999	*1*	793.1	0.73	0.00
		*2*	776.7	1538.65	8.48
		*3*	705.1	0.33	0.00
		*4*	672.2	3362.91	24.74
		*5*	589.5	304.37	2.91
Tectoquinone	0.0801	*1*	796.9	0.21	0.00
		*2*	744.3	0.00	0.00
		*3*	603.5	685.38	6.26
		*4*	585.8	491.85	4.77
		*5*	580.0	192.49	1.90
Variecolorquinone A	0.1044	*1*	814.2	17.18	0.09
		*2*	794.9	249.46	1.31
		*3*	742.9	3.07	0.02
		*4*	718.5	6278.35	40.43
		*5*	627.6	1759.58	14.85
Ventinone A	0.1251	*1*	941.5	1175.86	4.41
		*2*	764.6	7893.62	44.89
		*3*	762.5	32.07	0.18
		*4*	660.4	0.63	0.00
		*5*	658.3	3641.03	27.93
Versiconol	0.1138	*1*	838.2	194.61	0.92
		*2*	800.4	1.00	0.01
		*3*	713.5	4050.42	26.45
		*4*	685.2	4641.11	32.86
		*5*	646.7	4.83	0.04
Versiconol B	0.1126	*1*	819.4	217.49	1.08
		*2*	812.3	0.26	0.00
		*3*	718.6	5404.77	34.79
		*4*	702.6	2958.98	19.92
		*5*	657.4	3.41	0.03
Xanthopurpurin	0.0999	*1*	792.5	0.34	0.00
		*2*	760.1	1210.27	6.96
		*3*	700.5	0.16	0.00
		*4*	629.9	1112.89	9.32
		*5*	587.4	741.49	7.14
Ziganein	0.0624	*1*	789.2	35.40	0.19
		*2*	780.5	1.06	0.01
		*3*	754.4	4019.00	23.47
		*4*	699.3	0.01	0.00
		*5*	620.4	2207.56	19.07

### 2.2. Two–Photon Excitation Wavelengths

**Figure 2:**
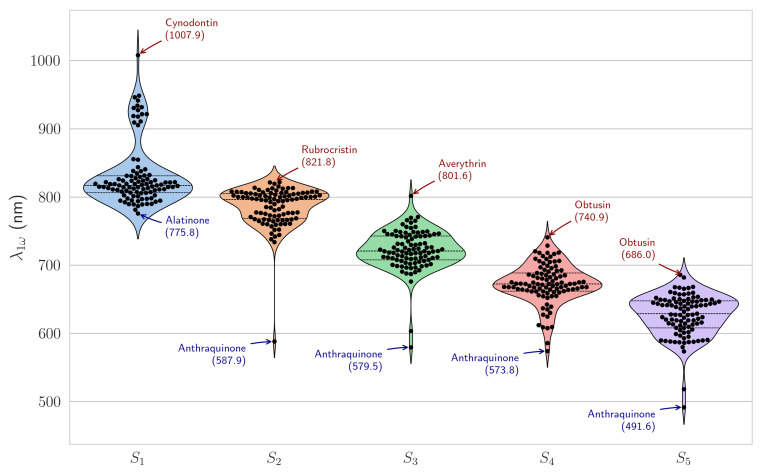
Violin plots showing the distribution of calculated $\lambda_{1\omega }$ for the five lowest singlet excited states. Horizontal lines indicate mean (dashed) and quartiles (dotted), and individual data points are overlaid.

The calculated energy of the single degenerate photon that is required for the two–photon excitation ( $\lambda_{1\omega }$ ) for all studied anthraquinones fall within the biological therapeutic window if S_0_→S_1_ are considered, which is particularly advantageous for potential biomedical applications such as two–photon microscopy and photodynamic therapy (Figure 2). $\lambda_{1\omega }$ exhibit a systematic blue–shift with increasing excited state energy, progressing from a mean of 831.7 nm for S_1_ to 626.3 nm for S_5_. Notably, the distributions show remarkably low variance (standard deviations of 29.4–46.3 nm), indicating that despite structural variations within the anthraquinone scaffold–including different hydroxylation patterns, methyl substitutions, and extended conjugation–the electronic transitions remain relatively consistent.

Several compounds exhibit particularly long $\lambda_{1\omega }$ for S_1_, with cynodontin reaching a maximum of 1007.9 nm. This red–shifted absorption can be attributed to cynodontin’s multiple hydroxyl substituents, which increase π-electron delocalization and lower the effective HOMO–LUMO gap, resulting in longer-wavelength transitions. Other compounds in this subgroup include austrocortinin (921.6 nm), catenarin (931.6 nm), demethoxyaustrocortirubin (921.5 nm), dermocybin (911.0 nm), dermorubin (946.2 nm), erythroglaucin (927.6 nm), fistulic acid (918.2 nm), helminthosporin (930.4 nm), islandicin (934.3 nm), pseudopurpurin (905.6 nm), purpurin (909.5 nm), quinizarin (918.9 nm), rubrocristin (948.8 nm), and ventinone A (941.5 nm). In contrast, the generic anthraquinone structure consistently exhibits the shortest $\lambda_{1\omega }$ across S_2_–S_5_ (587.9–491.6 nm), reflecting its minimal conjugation.

### 2.3. Two–Photon Absorption Cross–Sections

**Figure 3:**
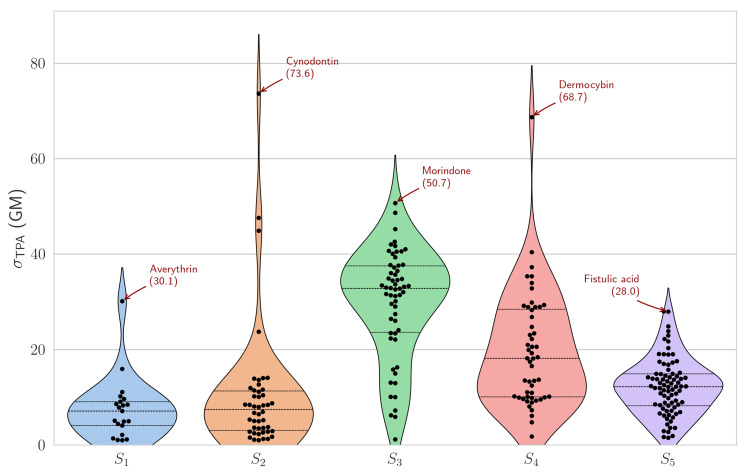
Violin plots displaying the distribution of calculated $\sigma_{TPA}$ for the five lowest singlet excited states. Only compounds with $\sigma_{TPA}$ > 1 GM are included. Horizontal lines indicate mean (dashed) and quartiles (dotted), and individual data points are overlaid.

Analysis of two–photon absorption cross–sections reveals pronounced state–dependent behaviour (Figure 3). Among the five excited states examined, S_3_ demonstrates optimal TPA efficiency, with 58 compounds (60% of the dataset) exhibiting cross–sections exceeding 1 GM, a mean value of 29.9 GM, and a relatively narrow distribution (standard deviation of 11.4 GM). The median cross–section for S_3_ (32.8 GM) significantly exceeds those of other states, suggesting that this excited state may benefit from electronic and structural features that enhance two-photon absorption, such as symmetry properties that make two-photon transitions more allowed and electronic configurations that give rise to stronger effective transition moments and resonance enhancement.

**Figure 4:**
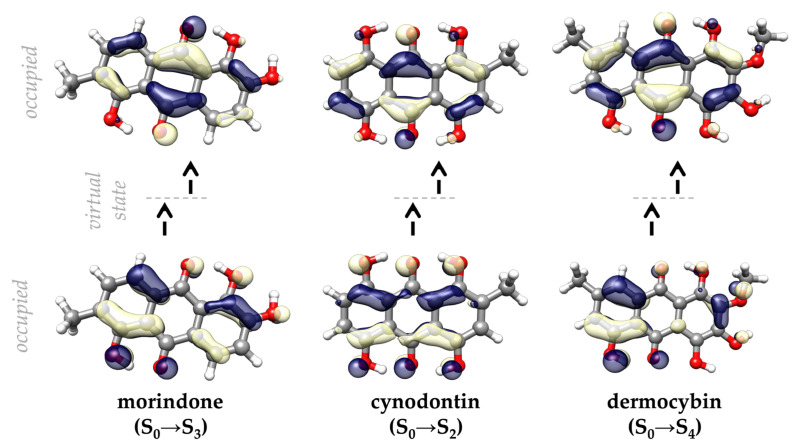
Natural transition orbitals for the states exhibiting the highest two–photon absorption cross–sections of morindone, cynodontin, and dermocybin.

Within the S_3_ manifold, morindone achieves a notable two–photon absorption cross–section of 50.7 GM. However, the largest TPA responses are observed for cynodontin (73.6 GM, S_2_) and dermocybin (68.7 GM, S_4_), suggesting that specific auxiliary substitution patterns lead to enhanced two-photon transition amplitudes in discrete excited states through increased effective transition dipoles and favourable charge-transfer character. These exceptional values approach those of purpose–designed synthetic chromophores while being derived from naturally occurring scaffolds. These excitations are characterized by exceptionally high configurational purity, involving the H–1 → L (86.5%) transition for morindone, the H–1 → L (94.2%) transition for cynodontin, and the H–2 → L (82.5%) transition for dermocybin. Natural transition orbitals (NTO) analysis (Figure 4) further confirms this, underlining the particle → hole contributions equal 94.7%, 96.6%, and 95.5%, respectively.

In the context of CC2 wavefunctions, this phenomenon indicates that the excited states are dominated by one–electron π→π* character, consistent with high configurational purity and strong transition moments. In these non–centrosymmetric systems, such “bright” single–excitation states are the primary drivers of TPA activity. In the dipolar excitation regime characteristic of these molecules, efficient two-photon transitions arise from both substantial electric transition dipole moments and significant changes in the permanent dipole moment upon excitation, which together enhance the overall two-photon transition strength.

The underlying physical origin of this enhancement is further elucidated by the transition dipole moment, which exhibits extreme anisotropy along the longitudinal (*x*) molecular axis. Specifically, the *xx*–component of the tensor reaches 280.0 a.u. for morindone, −380.7 a.u. for cynodontin and −299.2 a.u. for dermocybin, while the *yy* and *zz* contributions remain much lower. This pronounced tensor divergence—where the *xx* component is nearly two orders of magnitude larger than the transverse elements—indicates that the TPA response is governed by a highly directional and unobstructed redistribution of electron density.

In the case of cynodontin, the strategic 1,4,5,8–hydroxylation pattern effectively maximizes the electronic decoupling between the hole and particle NTOs and promotes intramolecular charge redistribution. By positioning electron-donating hydroxyl groups flanking the central carbonyl acceptor moieties, the molecule establishes an efficient donor–acceptor (push–pull) electronic architecture [15] that enhances π-electron delocalization and lowers the energy gap relevant to two-photon transitions. Enhanced charge-transfer character is well known to increase two-photon absorption efficiency by increasing transition dipole strengths and promoting favorable intermediate state coupling. This mechanism remains effective despite the symmetry-breaking perturbation of the C_2_–methyl group. While morindone and dermocybin also benefit from analogous charge-transfer enhancement, their substitution patterns do not achieve the same degree of longitudinal π-delocalization as the 1,4,5,8-scaffold, resulting in slightly attenuated TPA responses.

In contrast, S_1_ and S_5_ exhibit substantially lower TPA efficiencies. Only 21 compounds (22%) show S_1_ cross–sections above 1 GM (mean: 7.5 GM), whilst S_5_ displays moderate activity with 81 compounds exceeding the threshold (mean: 12.2 GM). The intermediate states S_2_ and S_4_ show variable behaviour, with 46 and 52 compounds, respectively, exceeding 1 GM, though their distributions are broader (standard deviation of 13.4 and 11.9 GM) and exhibit significant outliers.

### 2.4. Structure–Property Relationships

A systematic analysis was performed to elucidate the fundamental relationships governing the maximum two–photon absorption ( $\sigma_{TPA}^{max}$ ) within the studied dataset.

#### 2.4.1. Hydroxylation

The position of hydroxyl substituents influences the molecular symmetry, which in turn affects the TPA activity, dictating the brightness of specific transitions and their corresponding energies. The parent anthraquinone molecule (*D_2h_* symmetry) exhibits forbidden S_1_, S_3_, and S_5_ excitations because they do not have the same inversion parity as the ground state. Under electric-dipole two-photon selection rules in centrosymmetric systems, transitions are allowed only between states of equal inversion parity (g ↔ g or u ↔ u). It also possesses a very low $\sigma_{TPA}^{max}$ of 6.1 GM at 573.8 nm (S_4_). This baseline confirms that the unsubstituted centrosymmetric core exhibits a low TPA cross section because many low-energy transitions are symmetry-forbidden or weak under inversion-parity selection rules.

Hydroxyl substitution lowers the molecular point group (*D_2h_* → *C_s_* or *C_2v_*), relaxing inversion parity constraints that restricted two-photon transitions in the centrosymmetric parent and allowing for the mixing of electronic states that were previously forbidden. Hydroxylation at the 1,4–positions (quinizarin) acts as a regiochemical determinant that activates the S_1_ and S_5_ excitations while quenching S_2_. This para–substitution pattern (1,4–positions, both α to the carbonyl groups) induces a longitudinal dipole moment along the long axis of the core, which may facilitate NIR transitions and result in a $\sigma_{TPA}^{max}$ of 9.7 GM at 627.4 nm (S_4_). Further hydroxylation to form the 1,2,4–trihydroxy derivative (purpurin) maintains the same excitations as bright, slightly increasing $\sigma_{TPA}$ in the case of S_1_ and S_4_, while doubling it for S_5_ (9.3 GM at 608.1 nm).

Conversely, ortho (1,2) and meta (1,3) patterns favour the mid–energy manifolds. 1,2–dihydroxyanthraquinone (alizarin) displays a robust S_2_ transition, yielding a significantly improved $\sigma_{TPA}^{max}$ of 14.1 GM at 799.8 nm. This contrasts with the 1,3–dihydroxy derivative (xanthopurpurin), where the $\sigma_{TPA}^{max}$ is 9.3 GM at 629.9 nm (S_4_). In the 1,2,3–trihydroxy derivative (anthragallol), transition probability redistributes due to electronic coupling between adjacent substituents, resulting in a $\sigma_{TPA}^{max}$ of 11.6 GM at 764.9 nm (S_2_).

The most potent configurations involve peri–substitution (1,5 or 1,8), which maximises the two–photon transition tensor through enhanced polarisability along the long molecular axis: 1,5–dihydroxyanthraquinone (anthrarufin) achieves a high $\sigma_{TPA}^{max}$ of 23.7 GM at 752.1 nm (S_2_). The 1,8–hydroxylation pattern (danthron/chrysazin) offers the most effective response amongst dihydroxy derivatives, reaching a $\sigma_{TPA}^{max}$ of 29.0 GM at 730.1 nm (S_3_). The close proximity of the 1,8–hydroxyls to the central carbonyl facilitates intramolecular hydrogen bonding, which reduces conformational flexibility and may rigidify the molecular geometry. This trend culminates in 1,3,6,8–tetrahydroxyanthraquinone (rheoemodin), where the $\sigma_{TPA}^{max}$ reaches 31.7 GM at 699.8 nm.

#### 2.4.2. Alkylation

Alkylation of the anthraquinone core, primarily *via* methyl substitution, serves as a critical mechanism for tuning nonlinear optical properties. Beyond simple inductive enhancement of the π–system electron density, methyl groups act as symmetry–breaking perturbations that reshape the excited–state manifold, frequently inducing a redistribution of oscillator strength between electronic channels.

The most elementary evidence of this symmetry breaking is observed in the 2–methyl derivative (tectoquinone). In contrast to the parent anthraquinone, which displays a forbidden S_3_ and S_5_ transitions, tectoquinone exhibits both as bright states, with S_3_ linked with a $\sigma_{TPA}^{max}$ of 6.3 GM at 603.5 nm, while fading away S_2_. This confirms that even a single alkyl group on an otherwise unsubstituted scaffold is sufficient to break the core’s inversion symmetry, thereby relaxing the parity selection rules that previously hindered these transitions.

More sophisticated methyl–induced state redistribution is revealed when comparing 1,5–dihydroxy and 1,5–dihydroxy–3–methyl derivatives (anthrarufin and ziganein, respectively). In anthrarufin, TPA activity is distributed across the S_2_ (23.7 GM at 752.1 nm), S_4_ (18.2 GM at 609.2 nm), and S_5_ (17.43 GM at 518.2 nm) states. However, the addition of the 3–methyl group in ziganein suppresses S_2_ and S_4_ states, suppressing their cross–sections to values below 1.0 GM, whilst shifting the primary TPA activity to the S_3_ state, which exhibits a $\sigma_{TPA}^{max}$ of 23.5 GM at 754.4 nm. Simultaneously, the S_5_ response is enhanced (19.1 GM at 620.4 nm). This demonstrates that alkylation can function as a mechanism for selective redistribution, redirecting energy within the manifold without sacrificing absolute cross–section magnitude.

In the 1,8–dihydroxy series, the methyl group provides a consistent enhancement to the nonlinear response. Moving from danthron/chrysazin (29.0 GM at 730.1 nm in S_3_) to chrysophanol increases the $\sigma_{TPA}^{max}$ to 31.4 GM at 726.0 nm.

The synergistic coupling between alkylation and hydroxylation is highly sensitive to relative substituent positioning. For instance, emodin displays a $\sigma_{TPA}^{max}$ of 33.7 GM in the S_3_ manifold at 712.2 nm. In stark contrast, alatinone, in which one of the rings have a shifted oxygen–methyl pattern, displays a significantly lower $\sigma_{TPA}^{max}$ of 19.04 GM at 639.7 nm (S_3_), albeit renders S_1_ and S_2_ excitable. This discrepancy indicates that, for maximum TPA enhancement, the alkyl groups must be strategically aligned within the framework established by the hydroxyl groups.

The absolute performance peak for alkylated species is achieved in cynodontin. By combining the optimised 1,4,5,8–tetra–α–hydroxyl motif with strategic methyl placement, the molecule achieves an extraordinary $\sigma_{TPA}^{max}$ of 73.6 GM at 815.5 nm (S_2_).

#### 2.4.3. Alkoxylation

Conversion of hydroxyl groups to ethers (Ar–O–R) represents a sophisticated structural modification yielding some of the most dramatic TPA enhancements in the natural anthraquinone library. This alkoxylation effect stems from dual physical mechanisms: the enhancement of oxygen’s donor strength and, crucially, the elimination of intramolecular hydrogen bonding between α–substituents and the central anthraquinone carbonyls. By releasing the oxygen lone pairs from hydrogen–bonding constraints, the electronic coupling between the peripheral donors and the aromatic core is significantly amplified.

The most prominent evidence of this effect is found in the comparison between emodin and its monomethyl ether, physcion. In emodin, the maximum TPA cross–section is localised in the S_3_ state at 33.7 GM. Upon methoxylation of the 3–hydroxyl group to form physcion, the $\sigma_{TPA}^{max}$ surges to 41.0 GM at 711.3 nm (S_3_), representing an approximately 22% increase in brightness without a significant wavelength shift. This enhancement extends to the high–energy manifold, where physcion’s S_5_ state (15.7 GM) outperforms that of emodin (12.6 GM), demonstrating that alkoxylation provides enhancement across multiple transitions.

The impact of progressive alkoxylation is further elucidated by the obtusifolin/obtusins series. Obtusifolin exhibits a bifurcated TPA response with comparable activity in the S_2_ and S_4_ manifolds, yielding a $\sigma_{TPA}^{max}$ of 11.0 GM (S_4_). Alkoxylation of the C_8_ hydroxyl group and the introduction of –OCH_3_ substituents at C_6_ and C_7_ in chryso–obtusin lead to a more balanced distribution across the manifold, with S_3_, S_4_, and S_5_ ( $\sigma_{TPA}^{max})$ states all displaying cross–sections around 10.1–10.5 GM. Reverting the methylation at C_8_, as in obtusin, produces a cumulative substituent effect that results in a strong high–energy response, with the $\sigma_{TPA}^{max}$ reaching 17.3 GM at 686.0 nm (S_5_). Reverting another methylation (at C_6_) which is the case of aurantio–obtusin cause a substantial mid–range response, with a $\sigma_{TPA}^{max}$ of 12.7 GM (S_2_). Overall, these results suggest that while extensive alkoxylation may distribute oscillator strength across multiple electronic channels, optimal substitution patterns can significantly enhance the total nonlinear absorption relative to simpler hydroxylated analogues.

#### 2.4.4. Carbonylation

Introduction of aldehyde functionality onto the anthraquinone core introduces strong electron–withdrawing character that competes with the central quinone acceptor. This modification generally results in a blue–shift in the TPA response and a redistribution of oscillator strength towards higher–lying excited states, contrasting sharply with the long–wavelength activity seen in purely hydroxylated and alkylated derivatives.

Damnacanthal exemplifies this behaviour, exhibiting its $\sigma_{TPA}^{max}$ of 12.3 GM at 614.5 nm (S_5_). Its lower–lying states (S_1_ through S_4_) are significantly suppressed, with the S_3_ cross–section reaching only 1.2 GM. This indicates that the formyl group at the 2–position effectively suppresses donor–acceptor transitions to lower excited states, redirecting oscillator strength to higher–energy electronic channels. The C_1_–demethylated analogue, nordamnacanthal, shows a similar profile but with enhanced low–energy activity; whilst its $\sigma_{TPA}^{max}$ remains in the S_5_ state (13.8 GM at 635.5 nm), it also exhibits an activated S_2_ transition of 3.5 GM at 786.1 nm. This change from methoxy to hydroxyl at the 3–position restores some degree of lower–energy polarisation, albeit with a relative reduction in absolute brightness compared to non–formylated trihydroxy systems.

The impact of the formyl group is further nuanced for fallacinal. Structurally related to fallacinol but bearing an aldehyde at the 3–position, fallacinal achieves a formidable $\sigma_{TPA}^{max}$ of 37.6 GM at 720.3 nm (S_3_). While impressive, a direct comparison with its hydroxymethyl precursor, fallacinol ( $\sigma_{TPA}^{max}$ = 42.0 GM in S_3_) reveals that alternation of the side chain to a carbonyl results in an approximately 10.5% reduction in peak TPA efficiency and the near–total quenching of high–energy manifold activity (the S_5_ state drops from 17.0 GM in fallacinol to near–zero in fallacinal).

#### 2.4.5. Carboxylation

Introduction of carboxylic acid groups into the anthraquinone scaffold introduces electron–withdrawing character that significantly modulates the molecule’s electronic architecture. Despite the electron-withdrawing inductive effect of the carboxyl group, carboxylation primarily results in a consistent bathochromic shift in the TPA response—an effect that may arise from the participation of carboxylate oxygen lone pairs in π-conjugation or from favourable excited-state stabilisation through resonance structures. This red-shift is accompanied by maintained or slightly enhanced cross–section magnitude through improved charge–transfer character.

Direct comparison between rhein and its non–carboxylated analogue danthron (chrysazin) illustrates this spectral tuning property. Danthron exhibits a $\sigma_{TPA}^{max}$ of 29.0 GM at 730.1 nm (S_3_), but rhein displays a peak of 29.6 GM at 740.9 nm in the same manifold. This 10.8 nm red–shift, accompanied by a slight $\sigma_{TPA}$ increase, demonstrates that the carboxyl group acts as an auxiliary acceptor stabilising the excited state without disrupting the primary transition dipole established by the hydroxyl arrangement.

In more complex systems, carboxylation influences the distribution of oscillator strength across the manifold. Emodic acid maintains a peak S_3_ cross–section of 33.4 GM at 724.3 nm, nearly identical in magnitude to its parent compound emodin (33.7 GM), but with a 12.1 nm red–shift. This stabilisation is more pronounced in endocrocin, where the carboxyl group’s interaction with the trihydroxyl motif pushes the $\sigma_{TPA}^{max}$ to 40.6 GM at 722.9 nm (S_3_). This suggests that the –COOH group can synergistically interact with multiple donors to increase overall π–system polarisability.

The most potent carboxylated derivatives are found amongst methoxylated species. Parietinic acid (the carboxylic acid derivative of physcion) achieves an S_3_ $\sigma_{TPA}^{max}$ of 40.1 GM at 723.3 nm, with a robust secondary response in the S_5_ state (19.0 GM). Furthermore, carboxyl regiochemistry can force transitions between excited states, as seen in dermolutein. In this molecule, TPA activity migrates from S_3_ (characteristic of emodin–like systems) to the S_4_ manifold, reaching 35.4 GM at 716.6 nm. Similarly, dermorubin demonstrates the efficacy of combining carboxylation with extensive hydroxylation, yielding a bifurcated response with a $\sigma_{TPA}^{max}$ of 33.4 GM at 732.1 nm (S_3_) and 22.9 GM in the S_5_ state.

#### 2.4.6. Halogenation

Incorporation of halogen atoms (specifically chlorine) into the anthraquinone framework introduces significant inductive effects and increases molecular polarisability. Fragilin, the chlorinated analogue of physcione, provides a clear example of this electronic redistribution. Whilst physcion exhibits dominant S_3_ and S_5_ transitions, fragilin has the response split between the S_3_ (23.4 GM at 748.6 nm) and S_4_ manifolds, with the latter representing a $\sigma_{TPA}^{max}$ of 33.9 GM at 712.7 nm. This migration to the S_4_ state represents a significant shift in spectral distribution and a noticeable increase in the peak brightness compared to the parent molecule.

A similar pattern of manifold redistribution is observed in nalgiolaxin, a chlorinated and methoxylated derivative, which achieves a $\sigma_{TPA}^{max}$ of 35.4 GM in the S_4_ state (717.9 nm). In contrast, the non–chlorinated nalgiovensin concentrates its oscillator strength more efficiently in the S_3_ manifold, reaching a higher $\sigma_{TPA}^{max}$ of 45.2 GM at 716.5 nm.

#### 2.4.7. Nitrogen–Containing Derivatives

Introduction of nitrogen–containing functional groups, particularly in amide form, introduces a significant enhancement mechanism to the TPA properties of anthraquinones. The emodacidamide series, derived from the natural product emodic acid, demonstrates how the inclusion of an amide linkage can dramatically enhance charge–transfer character through the involvement of the nitrogen lone pair.

Emodacidamide A achieves a $\sigma_{TPA}^{max}\;$ of 41.7 GM at 719.9 nm (S_3_), representing a significant approximately 24% enhancement over the parent emodic acid (33.4 GM). This high efficiency is maintained across the series, with emodacidamide H exhibiting a $\sigma_{TPA}^{max}$ of 39.3 GM in the S_3_ state (720.1 nm). The amide group acts as a potent auxiliary donor, stabilising the charge–transfer excited states and increasing transition probability without inducing a significant shift in excitation energy. Additionally, these compounds show enhanced $\sigma_{TPA}\;$ in the S_5_ manifold, suggesting that the nitrogen–based donor provides a broader electronic contribution than oxygen–based donors alone.

#### 2.4.8. Multi–Substituent Synergy

The highest two–photon absorption cross–sections in the natural anthraquinone library are found in molecules that combine multiple substituent types—hydroxyl, alkyl, and extended side chains—in configurations that maximise the transition dipole moment across the entire aromatic system. These synergistic molecules often exhibit non–additive enhancements, where the final cross–sections far exceed the mere sum of individual functional group contributions due to the constructive interference of multiple charge–transfer pathways.

Averythrin stands out as a prominent example of this synergy. By combining a polyhydroxylated core with alkenyl chain, it activates the low–energy manifold to an extraordinary degree, achieving a significant NIR–I response with a $\sigma_{TPA}^{max}$ of 47.6 GM at 821.3 nm (S_2_) and a robust S_1_ transition (30.1 GM at 843.7 nm). Similarly, averantin—despite lacking a double bond in the side chain but possessing a related substitution pattern—exhibits a potent S_3_ transition of 31.4 GM (710.7 nm) and a significant S_4_ response of 22.2 GM. These molecules demonstrate that long–chain alkyl groups can act as auxiliary polarisable units, further extending the effective length of the transition dipole.

The performance peak is reached in dermocybin. This molecule represents a near–optimal arrangement of donor groups—four hydroxyls, one methoxy, and one methyl—which together create an exceptionally polarisable π–system. Dermocybin achieves a $\sigma_{TPA}^{max}$ of 68.7 GM at 717.9 nm (S_4_), while maintaining broad–spectrum activity across the S_1_ (7.2 GM) and S_2_ (8.1 GM) regions. The presence of hydroxyls, alkoxy, and alkyl groups effectively leads to cross–sections more than ten times greater than the parent anthraquinone.

### 2.5. Solvent Effects and Gas–Phase Limitations

A critical limitation of this study concerns the gas–phase treatment of TPA properties. Solvent effects on TPA cross–sections may arise primarily through two mechanisms: (1) direct electronic perturbation of the excited–state manifold, typically leading to modest red–shifts and cross–section changes, and (2) conformational effects, where solvent–induced geometry changes alter the electronic structure. For the rigid aromatic core of anthraquinone, conformational effects are expected to be minimal.

To address the former concern, aqueous–phase computations were performed for three previously studied compounds–aloe–emodin, soranjidiol, and rubiadin–using the COSMO implicit solvation model (Table 2). The results reveal substantial and non–systematic solvent effects that defy simple correction schemes. Whilst excitation wavelengths shift modestly (≤20 nm), cross–sections exhibit dramatic state–dependent variations: aloe–emodin’s S_3_ increases from 32.77 to 47.08 GM, yet rubiadin’s S_1_ surges from essentially zero to 10.79 GM, whilst its S_4_ collapses from 11.07 to 0.04 GM. Similarly, soranjidiol’s S_2_ decreases from 8.48 to 0.01 GM, while S_3_ increases from 0.00 to 41.06 GM.

These pronounced redistributions may stem from solvent–driven reordering of excited–state energies and modified coupling via intermediate states–phenomena inherent to the physics underlying two–photon absorption. Notably, the state with the largest cross–section can differ entirely between the gas phase and aqueous solution, undermining any assumption that gas–phase trends extrapolate reliably to solvated environments. Consequently, the gas–phase dataset functions chiefly as an internal reference for relative comparisons within a consistent computational protocol, whereas quantitative agreement with experiment requires explicit inclusion of solvent effects. Further investigations with broader datasets will be essential to clarify these discrepancies and provide more robust feedback.

## 3. Materials and Methods

### 3.1. Conformational Sampling

Each anthraquinone derivative was subjected to comprehensive conformational exploration using the Global Optimisation Algorithm (GOAT) [20] implemented in Orca (v.6.1.0) [21,22]. The XTB2 semiempirical tight–binding method [23] was employed to generate an ensemble of molecular conformers. This approach balances computational efficiency with sufficient accuracy for exploring the conformational landscape of these moderately sized aromatic systems. From the resulting ensemble, the most stable, energetically representative conformer was selected for subsequent high–level calculations.

### 3.2. Geometry Optimisation

Ground–state geometries were optimised using the TPSSh [24,25,26] meta–hybrid generalised gradient approximation functional (LibXC implementation [27]) combined with the def2–TZVP triple–ζ quality basis set [28]. Grimme’s D4 dispersion correction [29,30] was applied to account for London dispersion interactions, which are particularly important in extended aromatic systems. Harmonic vibrational frequency calculations confirmed the nature of all optimised structures as true minima on the potential energy surface (absence of imaginary frequencies).

### 3.3. Two–Photon Absorption

Non–linear optical properties were derived using the ricc2 module of Turbomole (rev. V7–9) [31] at the RI–CC2 (Resolution–of–Identity Coupled–Cluster Singles and Doubles) level of theory with the aug–cc–pVDZ basis set [32] and corresponding optimised auxiliary basis functions. The RI–CC2 method represents an optimal compromise between computational cost and accuracy for excited–state properties of medium–sized organic molecules, offering substantial improvements over time–dependent density functional theory for charge–transfer excited states whilst remaining computationally feasible for systematic studies [33]. All calculations were performed in the gas phase, as the applied methodology was found to be computationally prohibitive in implicit solvent models for datasets of this size.

Two–photon absorption cross–sections ( $\sigma_{TPA}$ ) were derived from the rotationally averaged transition strengths for linearly polarised light, $\langle \delta_{TPA}\rangle$ , following Equation (1):

(1) $$\sigma_{TPA}=\frac{N\pi^{3}\alpha a_{0}^{5}\omega^{2}}{c}\langle \delta_{TPA}\rangle g(2\omega ,\omega_{0},\Gamma )$$

Here, N = 4 represents the degeneracy factor appropriate for single–beam two–photon absorption experiments, α is the fine structure constant (≈1/137.036), $a_{0}$ is the Bohr radius (0.529177 Å), ω is the photon energy in atomic units, c is the speed of light, and $g(2\omega ,\omega_{0},\Gamma )\;$ is the normalised Gaussian lineshape function describing spectral broadening effects. A broadening parameter Γ of 0.1 eV (defined as the half–width at half–maximum) was employed, consistent with commonly adopted values in the literature for solution–phase measurements [7,34,35,36]. The computed $\sigma_{TPA}$ values were converted to macroscopic Göppert–Mayer units (1 GM = 10^−50^ cm^4^∙s∙photon^−1^) for ease of comparison with experimental data. For the comparison single–beam Lorentzian, double–beam Lorentzian, and double–beam Gaussian results, the outputs presented here should be multiplied by the factors 1.476^−1^, 1.355, and 2.000, respectively.

For each compound, the five lowest–energy singlet excited states (S_1_–S_5_) were considered, as these typically encompass the energetically accessible transitions relevant to bioimaging and photodynamic therapy applications.

### 3.4. Multi–Reference Diagnostics

To validate the appropriateness of the single–reference CC2 method for this diverse set of anthraquinone derivatives, the D_1_ diagnostic was evaluated for all ground–state wavefunctions. This diagnostic quantifies the contribution of single–electron excitations in the coupled–cluster wavefunction and serves as an indicator of multi–reference character that would compromise the accuracy of single–reference coupled–cluster methods [17].

## 4. Conclusions

This comprehensive RI–CC2 dataset suggest that focusing solely on the lowest excited state for photodynamic activity fundamentally misrepresents anthraquinone photophysics: the S_3_ manifold’s systematic superiority–both in wavelength absorption and cross–section magnitude–indicates that rational design strategies ought to prioritise this state rather than simply minimising excitation energies.

Natural transition orbital analysis confirms that the exceptional TPA responses of cynodontin, dermocybin, and morindone arise from highly directional charge–transfer mechanisms, as evidenced by spatial separation between hole and particle NTOs and extreme transition dipole anisotropy (longitudinal components reaching −380.7 a.u.); the excited states exhibit high configurational purity (82.5–94.2% single–excitation character), validating the single–reference treatment. The systematic structure–property investigation establishes clear design principles demonstrating that heavy–atom–free anthraquinones can achieve reasonable $\sigma_{TPA}^{max}$ values through rational functionalisation strategies.

The non–systematic solvent–induced redistributions of TPA properties across excited states present a critical challenge to computational screening workflows, as they invalidate simple correction schemes and demonstrate that even qualitative state ordering can reverse upon solvation. Consequently, this dataset functions primarily as a reference framework for identifying structural motifs that enhance intrinsic TPA propensity within a consistent theoretical treatment, whilst highlighting that careful solvent–involving research and experimental validation in physiologically relevant media remains indispensable for compounds flagged as promising candidates.

## Figures and Tables

**Table 2:** Comparison of gas–phase and aqueous–phase two–photon absorption properties for three previously studied anthraquinones.

Molecule	S*_i_*	Gas Phase			Water (COSMO)		
		$\lambda_{1\omega }$	δ_TPA_	σ_TPA_	$\lambda_{1\omega }$	δ_TPA_	σ_TPA_
Aloe–emodin	*1*	817.9	0.37	0.00	815.3	106.45	0.53
	*2*	799.4	87.38	0.45	784.7	0.53	0.00
	*3*	725.7	5192.03	32.77	735.3	7657.68	47.08
	*4*	672.9	0.40	0.00	675.3	0.42	0.00
	*5*	614.8	962.03	8.46	629.7	1609.31	13.49
Rubiadin	*1*	790.0	0.49	0.00	784.3	1996.30	10.79
	*2*	767.4	1471.25	8.30	769.6	0.71	0.00
	*3*	703.5	7.73	0.05	717.3	2661.16	17.19
	*4*	690.6	1588.35	11.07	693.0	6.01	0.04
	*5*	587.9	821.32	7.90	605.3	1455.29	13.20
Soranjidiol	*1*	793.1	0.73	0.00	790.8	1564.42	8.32
	*2*	776.7	1538.65	8.48	773.1	1.07	0.01
	*3*	705.1	0.33	0.00	710.0	6226.91	41.06
	*4*	672.2	3362.91	24.74	696.1	1.29	0.01
	*5*	589.5	304.37	2.91	606.3	544.70	4.93

## Data Availability

The raw data supporting the conclusions of this article will be made available by the author on request.
