# Correction: Spiegel, M. Two–Photon Absorption Properties and Structure–Property Relationships of Natural 9,10–Anthraquinones: A Curated RI–CC2 Dataset. *Int. J. Mol. Sci.* 2026, *27*, 87

**DOI:** 10.3390/ijms27021084

**Published:** 2026-01-22

**Authors:** Maciej Spiegel

**Affiliations:** Department of Organic Chemistry and Pharmaceutical Technology, Faculty of Pharmacy, Wroclaw Medical University, Borowska 211A, 50–556 Wroclaw, Poland; maciej.spiegel@umw.edu.pl

In the original publication [1], the acknowledgment of PLGrid was missing. The corrected text is as follows: “Acknowledgments: The author gratefully acknowledges Polish high-performance computing infrastructure PLGrid (HPC Center: ACK Cyfronet AGH) for providing computer facilities and support within computational grant no. PLG/2025/018861.” The author states that the update is an acknowledgment and the scientific conclusions are unaffected. This correction was approved by the Academic Editor. The original publication has also been updated.

## References

[1] Spiegel M. (2026). Two–Photon Absorption Properties and Structure–Property Relationships of Natural 9,10–Anthraquinones: A Curated RI–CC2 Dataset. Int. J. Mol. Sci..

